# Microbiological Analysis of Necrosols Collected from Urban Cemeteries in Poland

**DOI:** 10.1155/2015/169573

**Published:** 2015-08-02

**Authors:** Ireneusz Całkosiński, Katarzyna Płoneczka-Janeczko, Magda Ostapska, Krzysztof Dudek, Andrzej Gamian, Krzysztof Rypuła

**Affiliations:** ^1^Laboratory of Neurotoxicology and Environmental Diagnosis, Faculty of Health Science, Wroclaw Medical University, 51-618 Wroclaw, Poland; ^2^Division of Infectious Diseases and Veterinary Administration, Department of Epizootiology and Clinic of Birds and Exotic Animals, Faculty of Veterinary Medicine, Wroclaw University of Environmental and Life Sciences, 50-366 Wroclaw, Poland; ^3^Department of Logistics and Transport Systems, Faculty of Mechanical Engineering, Wroclaw University of Technology, 50-371 Wroclaw, Poland; ^4^Department of Immunology of Infectious Diseases, Institute of Immunology and Experimental Therapy, 53-114 Wroclaw, Poland

## Abstract

Decomposition of organic matter is the primary function in the soil ecosystem, which involves bacteria and fungi. Soil microbial content depends on many factors, and secondary biological and chemical contaminations change and affect environmental feedback. Little work has been done to estimate the microbiological risk for cemetery employees and visitors. The potential risk of infection for people in the cemetery is primarily associated with injury and wound contamination during performing the work. The aim of this study was to analyze the microbiota of cemetery soil obtained from cemeteries and bacterial composition in selected soil layers encountered by gravediggers and cemetery caretakers. The most common bacterial pathogens were *Enterococcus* spp. (80.6%), *Bacillus* spp. (77.4%), and *E. coli* (45.1%). The fungi *Penicillium* spp. and *Aspergillus* spp. were isolated from 51% and 6.4% of samples, respectively. Other bacterial species were in the ground cemetery relatively sparse. Sampling depth was not correlated with bacterial growth (*p* > 0.05), but it was correlated with several differences in microbiota composition (superficial versus deep layer).

## 1. Introduction

Soil microbial content depends on many factors, and changes therein result from secondary biological and chemical contamination. The soil microbiota is affected by water content; amounts of mineral and organic substances; soil structure, composition, and degree of acidity; and gas-phase reactions occurring in soil [1]. Exogenous organic matter penetrates into soil in the form of secretions, excretions, and bacteria from dead animals and humans. Secondary biological contaminants also include manure and human sewage, household and farms beheaded, and precipitation washed from areas inhabited by humans and industrial environments [2, 3].

The decomposition of organic matter, which involves primarily bacteria and fungi, is fundamental for the functioning of the soil ecosystem. Soil decomposition contributes to a high degree of heterogeneity in physical, chemical, and/or biological composition [4]. Bacteria in soil are classified in two groups: autochthonous, referring to microorganisms adapted to the presence of minimal nutrients (*Arthrobacter* spp.,* Azotobacter* spp.,* Clostridium* spp.,* Nitrobacter* spp.,* Nitrosomonas* spp.,* Pseudomonas* spp.,* Serratia* spp.,* Bradyrhizobium* spp.,* Mesorhizobium* spp.,* Rhizobium* spp.,* Sinorhizobium* spp.,* Acidithiobacillus* spp.,* Desulfovibrio* spp., and* Thiobacillus* spp.) and zymogenic, encompassing microorganisms showing rapid growth only after the introduction of highly concentrated nutrients (*Bacillus* spp.,* Corynebacterium* spp.,* Escherichia coli*,* Proteus* spp., and thermophilic microorganisms) [5, 6].

A 1998 World Health Organization report described the potential impacts of cemeteries on the environment and human health, focusing on soil decomposition and soil and groundwater contamination. The authors pointed out that little research had examined cemetery-related sources of environmental contamination. In the following years some studies dealing with this matter were published [7]. An analysis of cemetery impacts on groundwater contamination conducted in Portugal in 2000-2001 involved hydrological and geographic surveys of cemeteries that took differences in lithological conditions into account [8].* E. coli*, including the O157:H7 serotype,* Campylobacter* spp.,* Salmonella* spp.,* Listeria* spp.,* Listeria monocytogenes*, and* Mycobacterium tuberculosis* have been isolated from cemetery environments [9–11].

The aim of this study was to analyze the microbiota of cemetery soil obtained from cemeteries in the region of Lower Silesia, Poland. Microbiota composition in selected soil layers encountered by gravediggers and cemetery caretakers was evaluated.

## 2. Materials and Methods

The Ethical Committee for Animal Experiments in Wrocław, Poland, approved this study and all samples were collected in accordance with the research protocol that is accepted.

### 2.1. Research Area

**Figure 1:**
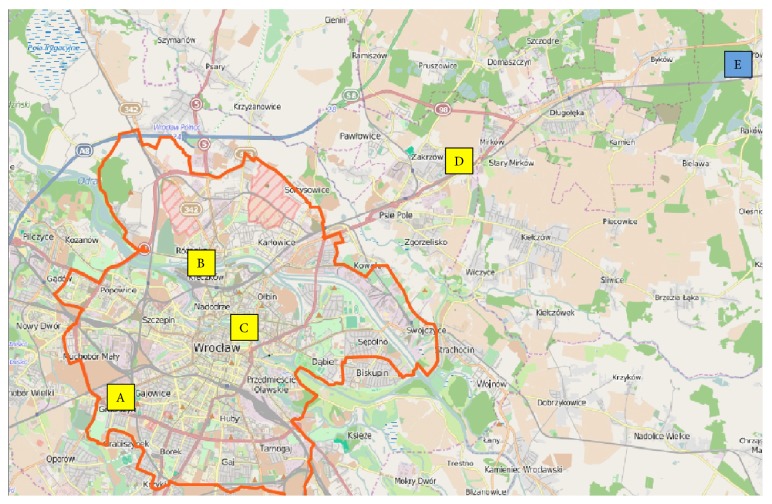
Location of the cemetery in the city of Wrocław; the yellow square indicates cemeteries in the metropolitan city of Wroclaw (A–D); the cemetery in Olesnica is highlighted in blue (E).

Samples were obtained from five urban necropolises: four in Wroclaw: Grabiszynski municipal cemetery: A, Osobowice municipal cemetery: B, municipal cemetery at the Bujwida Street: C, and municipal cemetery at the Kiełczowska Street: D, and one municipal cemetery in Oleśnica at the Polish Army Street: E (Figure 1). Cemeteries A–C were established in the 19th century (1881, 1867, and 1866, resp.), cemetery E has been operating since about 1926, and cemetery D, a municipal cemetery built in the postwar period, has held burials since 1996. All of these cemeteries continue to accept burials; in accordance with current Polish law [12], graves can be reused after 20 years if no person objects and the burial fee has not been paid. All cemeteries in the study sample are located in the same climate transition zone of the clearly temperate climate (dominated by oceanic influences), typical of Lower Silesia. The average yearly temperature hovers in the area of 8.5°C. The average amount of rainfall is 500–620 millimeters, with its maximum in July and minimum in February. The snow layer disappears after 45 days. Similar to west part/side of Poland, appearing winds are westerly and south-westerly.

### 2.2. Selection of Samples

**Figure 2:**
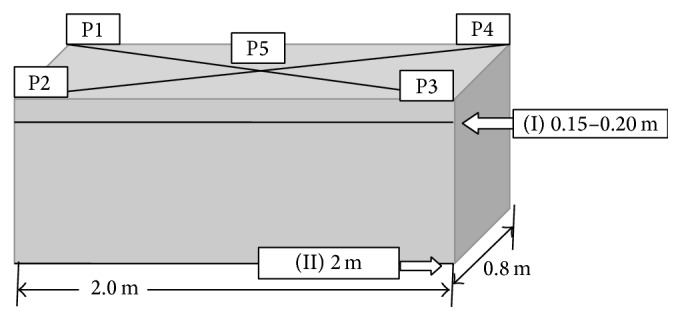
The diagram of sampling for analysis from one place of burial: on each of two depths (I, II), in each case the soil collected at the points P1–P4 (the corners of the grave) and P5 (a point defined at the intersection of the diagonals).

Samples for microbiological examination were collected from a total of 155 burial sites (A, *n* = 45; B, *n* = 20; C, *n* = 30; D, *n* = 35; E, *n* = 25) between summer 2013 and spring 2014. Each site was sampled only once. Soil samples were collected during simple grave preparation (reused places after 20 years) using 150 mL containers (Medlab, Poland). At two depths (0.15–0.20 m and 2 m), 200 g soil was taken from each of five points (four grave corners P1–P4 and center P5, identified by the intersection of diagonal lines from the corners; Figure 2). Samples were packed for shipping and transported by automobile to the EPI-Vet Diagnostic Laboratory, Faculty of Veterinary Medicine, Wroclaw, for microbial analysis. The laboratory implements a quality management system (ISO/IEC 17025:2005 + API:2007 + AC:2007).

### 2.3. Microbiological Analysis

Samples from P1–P5 were pooled at the laboratory for each depth, and the occurrence of microorganisms at the two depths was compared (comparison in the total sample per grave and per cemetery). All pooled samples from each depth were initially flooded with sterile PBS (IITD, Poland) in the volume ratio 1 : 1 and then with the sterile loop (10 *μ*L of the diameter, Sterbios, Poland) the material was seeded in agar with 5% horse blood and other media. The presence of aerobic and anaerobic gram-positive and gram-negative cocci and rods was evaluated. McConkey agar with crystal violet was used to detect gram-negative oxygen bacteria of the family Enterobacteriaceae, Mannitol Salt agar was used to identify growth of* Staphylococcus* spp., Enterococcosel agar was used for* Enterococcus* spp., and ORIE chromogenic substrate was used to detect bacteria in the family Enterobacteriaceae and gram-positive cocci. Sabouraud agar supplemented with chloramphenicol and gentamicin was used to detect growth of yeasts and molds. All culture media were supplied by GRASO Biotech (Poland). Bacterial preparations were incubated for 2 days at 37°C and samples used to detect fungi were incubated for 7 days at 25°C. Microorganism identification was based on morphological characteristics of the colony, gram staining, and biochemical characteristics, as defined using ENTEROtest 16 (Erba La Chema, Czech Republic). Fungal identification was based on the examination of direct preparations in saline under an optical microscope (BIOLAR C; CB, Poland) at 20 Å ~ magnification. Bacteria colonies were classified as very large (>10^4^ colony forming units (CFU)/mL), large (10^3^–10^4^ CFU/mL), or few (<10^3^ CFU/mL).

### 2.4. Statistical Analysis

The Pearson chi-squared test (two-tailed, significance level = 0.05) was used to compare proportions and cross table analysis was performed using Statistica 10.0.0 software (StatSoft).

## 3. Results

Seven genera of bacteria (*Bacillus* spp. (*Bacillus megaterium* and* B. cereus*),* Enterococcus* spp. (including* Enterococcus faecalis*),* Escherichia* spp., including* E. coli*, the* Klebsiella-Enterobacter-Serratia* (KES group), and* Staphylococcus* spp. including* Staphylococcus epidermidis* and other coagulase-negative staphylococci (CNS)) and two genera of fungus (*Penicillium* spp. and* Aspergillus* spp.) were isolated from cemetery soil samples. The most common pathogens were* Enterococcus* spp. (80.6%) and* Bacillus* spp. (77.4%). All bacterial species except* E. coli* (45.1%) were in the ground cemetery relatively sparse at 6.4% (*B. cereus*,* B. megaterium*,* E. faecalis*,* S. epidermidis*, and CNS).* Penicillium* spp. and* Aspergillus* spp. were isolated from 51% and 6.4% of samples, respectively. The results of microorganism analyses are shown in Table 1.

**Figure 3:**
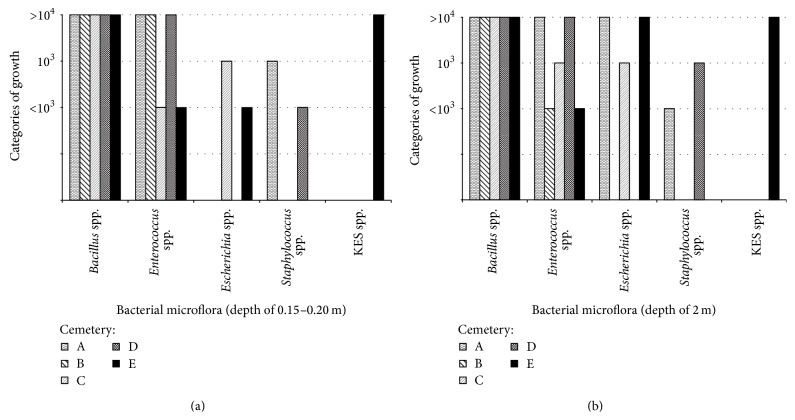
(a) The presence of bacterial microflora in the soil of burial at a depth of 0.15–0.20 m collected from the five necropolises (A–E). (b) The presence of bacterial microflora in the soil of burial at a depth of 2.0 m collected from the five necropolises (A–E). BS:* Bacillus* spp., EnS:* Enterococcus* spp., EsS:* Escherichia* spp., KES:* Klebsiella* spp.,* Enterobacter* spp., and* Serratia* spp., StS:* Staphylococcus* spp.

Figure 3 shows the bacterial growth levels of the two soil layers according to microorganisms identified.* Bacillus* spp. showed abundant growth (>10^4^ CFU/mL) in samples from all cemeteries, independent of sampling depth. Growth of* Enterococcus* spp. ranged from <10^3^ to >10^4^ CFU/mL in both soil layers. The growth of* Escherichia* spp., predominantly* E. coli*, was greater in samples from the deep soil layer (from 10^3^ to >10^4^ CFU/mL) than in those from the superficial layer (<10^3^ to 10^3^). The KES group was identified in samples from only one grave (at cemetery E), with >10^4^ CFU/mL observed in samples from both soil depths.

**Figure 4:**
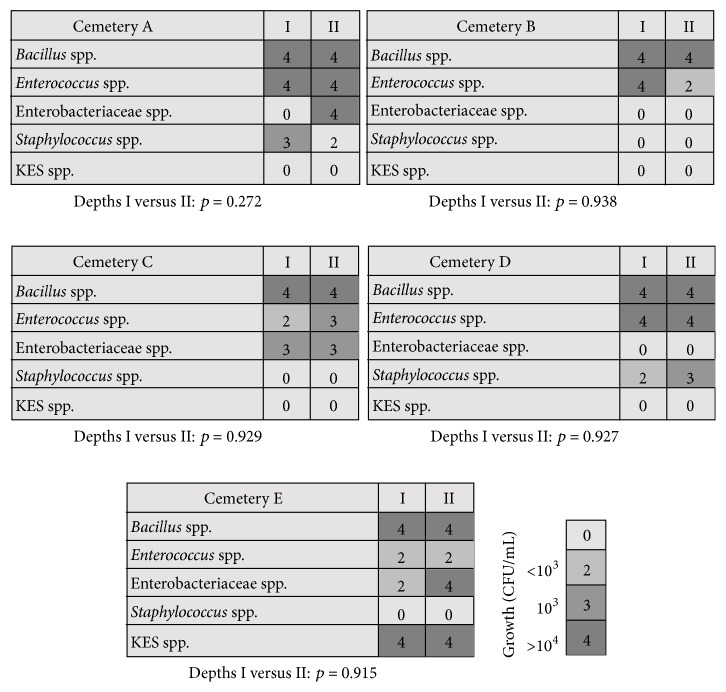
Microbial growth on five cemeteries including the depth and space sampling.

*Staphylococcus* spp. was identified at only two sites (cemeteries A and D); the growth of bacteria from cemetery A with <10^3^ CFU/mL was observed in samples taken from both soil depths, whereas in cemetery D the superficial layer generated less growth (<10^3^ CFU/mL) than the deep layer (10^3^–10^4^ CFU/mL). Three genera were predominant at all cemeteries:* Bacillus* spp.,* Enterococcus* spp., and* Escherichia* spp.* Penicillium* spp. was identified in samples from four of the five (80%) cemeteries. Sampling depth was not correlated with bacterial growth (*p* > 0.05; Figure 4), but it was correlated with several differences in microbiota composition (superficial versus deep layer:* Bacillus* spp. versus* Enterococcus* spp. (*p* = 0.021),* Bacillus* spp. versus KES group (*p* = 0.036),* Bacillus* spp. versus* Staphylococcus* spp. (*p* = 0.002), and* Enterococcus* spp. versus KES group (*p* = 0.019)).

## 4. Discussion

Research on cemetery soil emerged in the second half of the 20th century, accompanied by the introduction of the term “necrosol.” Subject to transformation of the soil profile, this layer does not exceed a depth of 0.2 m [13]. International research on necrosols has focused on their physicochemical properties, primarily phosphorus, nitrogen, and organic carbon contents [14, 15]. The groundwater in the vicinity of cemeteries showed increased concentrations of intestinal flora, ions, and amino acids, such as putrescine and cadaverine. The composition of the air in these environments is characterized by increased concentrations of unstable gases, such as phosphine and ethylene. Wax formation has also been observed in necrosols [16].

In Poland, according to the 7 March 2008 Regulation of the Minister of Infrastructure regarding cemeteries, graves and other burial sites of human remains and debris must be below ground and hold one individual casket of the dimension of 2 m × 1 m × 1.7 m (excluding children and family graves). This provision does not apply to cemeteries, graves, and other burial sites existing at the time of the regulation's establishment. The location of cemeteries established in the last two centuries did not account for intense urban development, which has led to a shortage of burial plots and permitted reuse of graves for which fees have not been paid after 20 years [17].

Little work has been done to assess the microbiological risk for cemetery employees and visitors. Knight and Dent [18] demonstrated that groundwater near burial sites contained high concentrations of* Pseudomonas aeruginosa*. Using the decomposition of fats by enzymes, Forbes et al. [19] indirectly showed the presence of* Clostridium perfringens* in necrosols.

However, microbiological analysis provided only supporting evidence in that study. The context of potential cemetery-related risks, that is, with respect to soil depth, has not been examined. The impacts of soil microbe profiles should be assessed with consideration of human contact, as in the present study. Moreover, analyses of groundwater contamination in/and around cemeteries does not allow confrontation with results achieved in Polish scientific study, as they have been performed in a different geographic location, also to each other [13–15].

The potential risk of infection for cemetery workers is associated primarily with injury and wound contamination during performance of the work. Lesser risks are associated with accidental microorganism ingestion, inhalation, or transfer to the mucous membranes of the eyes [20].

Live isolates from human tissue belong to the most common taxonomic groups, such as* Streptococcus* spp.,* Staphylococcus* spp.,* Clostridium* spp.,* Bacillus* spp., and* Lactobacillus* spp. [7]. The microbiota profiles of cemetery soil samples tested in the present study were similar to this ante mortem human microbial composition, with the addition of the KES group and* Aspergillus* spp. and* Penicillium* spp. fungi. This composition largely reflects the decomposition of human remains, as well as the flora and fauna of the burial sites. A limitation of the study is that the risk of contact with cemetery soil was not assessed, as not all genera of isolated microorganisms could be identified based on their biochemical properties. Pathogenic and commensal bacteria were identified.

The results of this study highlight the need for microbial analysis of necrosols using more sensitive laboratory techniques, such as polymerase chain reaction assays; however it would increase the cost of research. The level of some risks posed by identified species of bacteria in the analyzed soil layers could not be avoided.* Bacillus cereus* and* B. megaterium* may be primary causes of wound infection [6, 21].* B. cereus* is also considered to be the primary pathogen of food poisoning and eye infection, and it participates in progressive pneumonia and sepsis in infections of the nervous system [6, 22]. In recent years, an increased interest of* Enterococcus* spp. has been noted and the presence of bacteria of this genera has been confirmed within many infectious endogenous pathogenic bacteria isolates;* E. faecalis* is the dominant species (57%) in clinical cases and was also detected in the analyzed necrosol samples. The presence of this isolate is important in the context of possible injury-related contamination due to contact with cemetery soil. This pathogen produces hyaluronidase, an enzyme that depolymerizes mucopolysaccharides of connective tissue, which may facilitate the spread of bacteria and toxins [23]. Detailed characterization, which is not encompassed by the present research, also requires determination of the potential virulence of* E. coli* isolates from each grave and depth. This bacterium is the third most frequently isolated (after* S. aureus* and* P. aeruginosa*) from wounds and skin and soft tissue infections (SSTIs). These infections are often self-limiting, but some require antibiotic treatment and even hospitalization [24].* Staphylococci* were detected and identified in only two cases in the present study, but* S. intermedius* and CNS are known to cause many conditions, such as wound infections, skin abscesses, and inflammatory changes in joints [25]. The KES group comprises opportunistic organisms that commonly cause hospital infections related to manipulation in the respiratory tract (tracheostomy, inhalation) and catheterization.* Klebsiella* spp. rarely cause SSTIs, but cases of infection in the extremities, accompanied by purulent processes, gas formation, and metastatic lesions, have been reported [26]. Nail infection involving* Klebsiella* has also been reported [27]. Amin et al. [3] describe* Enterobacter* as accompanying SSTI. Skin lesions such as granulomas, fasciitis, lumps, abscesses, and ulcers caused by* Serratia* spp. are prevalent in elderly and immunocompromised individuals; however infections cannot be ruled out also in young and immunocompetent people [28].

Two genera of fungi were detected in this study.* Aspergillus* spp. and* Penicyllium* spp.* Aspergillus* spp. are common in the environment (in soil, food, air, water, decomposing plant, and animal materials) and* Penicillium* spp. cause infection, most commonly through the inhalation of spores. Like most fungi, these species are sensitizing, potentially causing asthma, allergic rhinitis, and atopic dermatitis [20]. The most serious disease caused by fungi of the genus* Aspergillus* spp. is pulmonary aspergillosis [29]. The exposure of different groups of people to cemetery soil microbiota should also be assessed. Microorganisms abound in different layers of the soil profile, and the frequency of contact is almost certainly greater among cemetery workers than among regular visitors. Further research in cooperation with human medicine and hospitals will be focused on the epidemiological aspects of human infections, because there are no literature data which currently record the place of infection (including cemetery), site of infection, and its correlation with developed disease stages.

## Figures and Tables

**Table 1:** The number and frequency (%) of isolations of individual bacteria and fungi in the ground of the cemetery, collected at the depths of 0.15–0.20 m (I) and 2 m (II) in the five necropolises (A–E)^*∗*^.

Pathogens	Cemetery									
	A		B		C		D		E	
	*n* = 45		*n* = 20		*n* = 30		*n* = 35		*n* = 25	
	I	II	I	II	I	II	I	II	I	II
*Bacillus* spp.	30	30	20	20	30	30	25	25	25	25
	67%	67%	100%	100%	100%	100%	71%	71%	100%	100%
*Bacillus cereus *	10	10	nd	nd	nd	nd	nd	nd	nd	nd
	22%	22%	0%	0%	0%	0%	0%	0%	0%	0%
*Bacillus megaterium *	10	10	nd	nd	nd	nd	nd	nd	nd	nd
	22%	22%	0%	0%	0%	0%	0%	0%	0%	0%

*Enterococcus* spp.	30	30	20	20	25	25	35	35	25	25
	67%	67%	100%	100%	83%	83%	100%	100%	100%	100%
*Enterococcus faecalis *	10	10	nd	nd	nd	nd	nd	nd	nd	nd
	22%	22%	0%	0%	0%	0%	0%	0%	0%	0%

Enterobacteriaceae spp.	25	25	5	5	15	15	5	5	25	25
*Escherichia coli *	56%	56%	25%	25%	50%	50%	14%	14%	100%	100%

KES group	nd	nd	nd	nd	nd	nd	nd	nd	20	20
*Serratia megaterium *	0%	0%	0%	0%	0%	0%	0%	0%	80%	80%

*Staphylococcus* spp.	10	10	nd	nd	nd	nd	10	10	nd	nd
	22%	22%	0%	0%	0%	0%	29%	29%	0%	0%
*Staphylococcus epidermidis *	10	10	nd	nd	nd	nd	nd	nd	nd	nd
	22%	22%	0%	0%	0%	0%	0%	0%	0%	0%
CNS	nd	nd	nd	nd	nd	nd	10	10	nd	nd
	0%	0%	0%	0%	0%	0%	29%	29%	0%	0%

*Penicillium* spp.	30	30	20	20	nd	nd	10	10	20	20
	67%	67%	100%	100%	0%	0%	29%	29%	80%	80%

*Penicillium* spp.	nd	nd	nd	nd	nd	nd	10	10	nd	nd
	0%	0%	0%	0%	0%	0%	29%	29%	0%	0%

^*∗*^Bacterial and fungal systematic given at the most detailed taxonomic level.
